# The effect of socioeconomic factors on quality of life of elderly in Jaffna district of Sri Lanka

**DOI:** 10.1371/journal.pgph.0000916

**Published:** 2022-08-31

**Authors:** Sathees Santhalingam, Sivayogan Sivagurunathan, Shamini Prathapan, Sivapalan Kanagasabai, Luxmi Kamalarupan

**Affiliations:** 1 Department of Nursing Faculty of Allied Health Sciences, University of Jaffna, Jaffna, Sri Lanka; 2 Department of Community Medicine Faculty of Medical Sciences, University of Sri Jayewardenepura, Nugegoda, Sri Lanka; 3 Department of Physiology Faculty of Medicine, University of Jaffna, Jaffna, Sri Lanka; Weill Cornell Medicine - Qatar, QATAR

## Abstract

Globally, the proportion of the elderly is increasing. In comparison to other Southeast Asian countries, Sri Lanka’s population is rapidly aging. The elderly are a vulnerable age group that requires special attention to live a long and healthy life. As, there was a scarcity of data on the elderly’s quality of life, studying the level of quality of life and the associated factors of the elderly in the Jaffna district will provide insight into how to plan interventions to improve the elderly’s overall well-being in Jaffna District and Sri Lanka as well. The study aimed to determine the quality of life of the elderly in the Jaffna district of Sri Lanka and to study the association of socioeconomic factors with the quality of life. This cross-sectional study was conducted among 813 community-dwelling elderly in the Jaffna District of Sri Lanka. Socio-economic characteristics were recorded by way of a structured questionnaire. The WHOQOL-Bref questionnaire was used to assess quality of life in four domains: physical health, psychological, social participation and the environment. The statistical Package of Social Science Software (SPSS) version 21 was used to analyse the data. Univariate, bivariate, and multivariate analyses were applied, p-value less than 0.05 was considered statistically significant. Among the four QOL domains, the mean (SD) score for an environmental domain was (12.1±2.1), (12.0±2.8) for the psychological domain, (11.8±2.3) for the physical health domain, and (10.1±3.0) for the social relationship domain. Factors significantly associated with all domains of QOL included marital status, level of education, living arrangement, employment, level of income, income adequacy and ownership of the house. Furthermore, age, sex, religion, number of children, and presence of monthly income, were significantly associated with at least one domain of QOL of the elderly. According to these findings, the QOL of the elderly in the Jaffna district of Sri Lanka seems low. And it was associated with multiple socio-economic factors. Interventions to improve the QOL of the elderly are anticipated with a higher emphasis on social relationship for the elderly.

## Introduction

Nowadays the elderly population is increasing globally, and it is a cause of concern. Longer life expectancy, low fertility rates, remarkable public health programs, and breakthroughs in medicine and health care are credited to this occurrence. In 1950, only 8% of the world’s population was over the age of 65; by 2000, that number had risen to 10%. [1]. Furthermore, it was predicted that the global elderly population would reach 22% in 2050. Therefore, one-fifth of the world’s population will be 60 or older in the near future [2]. The optimal Quality of life of the elderly is vital to enjoying the longevity of humankind.

The World Health Organization defines the quality of life as "an individual’s perception of their position in life in the context of the culture and value system in which they live and in relation to their goals, expectations, standards, and concerns." [3]. It is a multidimensional evaluation of a person’s ability concerning their physical, mental, social, and environmental health that can reflect a person’s overall well-being and is the most important indicator of a healthy life.

Health refers to an individual’s ongoing physical, emotional, mental, and social ability to cope with his or her surroundings. Therefore, the socioeconomic status of an individual plays a major role in their health and overall QOL. Numerous studies have been undertaken to examine the relationships between socio-economic characteristics (gender, age, marital status, education, and income) and quality of life in older people.

The global share of the elderly population is highest among developing countries [4]. Developing countries face special challenges with population aging and demographic shifts as their economic growth is low compared to the developed countries.

Sri Lanka is one of the developing countries with a rapidly increasing elderly population [5, 6]. Also, Sri Lanka has the highest proportion of elderly people among the Southeast Asian countries both today and in the future predictions [7]. In the year 2000, one in ten Sri Lankans was reported to be elderly. Furthermore, by 2010, this percentage had risen to 1 in 5, and it is anticipated to rise to one-fourth by 2030 [6, 8]. In addition, between 2000 and 2030, Sri Lanka’s median age is predicted to rise from 26.9 to 39.2 years [9]. The demographic shift should be managed by implementing policies to enhance the well-being of the elderly. The policies should give special emphasis to the districts that have the highest proportion of elderly people.

Apart from the effect of population aging on the country, aging and age-related changes pose greater challenges to the wellbeing of the elderly. Aging is the multifaceted, ongoing degradation of a person’s organ systems and tissue that is complex, inexorable, and unavoidable [10]. Individuals’ natural functionality is affected by aging. As a result, when compared to other age groups, the elderly are at a higher risk of a variety of physical and psychological difficulties. Musculoskeletal difficulties, respiratory disorders, and gastrointestinal and cardiovascular problems are the most common difficulties [3]. Further, the most prevalent health concerns reported by the aged in Sri Lanka include hypertension, diabetes mellitus, vision and hearing impairments, arthritis, and asthma [11]. Aside from the health problems mentioned above, many elderly people are also dealing with various socio-economic problems. Inevitably Physical health problems in the elderly with socio-economic problems together can have a significant impact on their wellbeing. Therefore, it is well understood that aging has a direct and indirect impact on the quality of life (QOL) of the elderly. Determining the QOL of the elderly and studying the association of socio-economic factors will be the initial steps to improving the wellbeing of the elderly population.

Only a small number of studies have been undertaken in Sri Lanka to examine the elderly’s quality of life in Sri Lanka. Jaffna District is one of Sri Lanka’s 25 administrative districts. It has a greater proportion of the elderly population (14.1%) than the rest of Sri Lanka (12.4%) [8, 12]. The majority of the QOL studies have focused on the southern area of the country and the Sinhala Buddhist community. As a result, there is a scarcity of information specific to the elderly in the Jaffna community. But Thanujanan et al (2016) demonstrated that the quality of life of the elderly of Jaffna District was determined to be moderate by using the OPQOL scale [13].

According to previous studies, there are many significant connections between quality of life and the socio-demographic and economic characteristics of the elderly. The goal of the study is to determine the quality of life and the association of socioeconomic factors among the elderly population residing in the Jaffna district of Sri Lanka and to make recommendations to improve the quality of life of the elderly who live in Jaffna district and Sri Lanka as well.

## Materials and methods

### Study samples and procedures

People over the age of 60 who lived in the Jaffna District were included in this cross-sectional descriptive study. The study excludes institutionalized elderly people who make up about 0.45% of Jaffna’s elderly population [14] as their living arrangements and care patterns differ from those of non-institutionalized elderly people.

In a recent study, the prevalence of self-reported QOL was 48% [15]. Based on a prevalence of 48%, a precision of 0.05, and a confidence level of 95%, the sample size required was calculated to be 384 using the Daniel formula [16]. It was increased to roughly 880 to account for the 15% nonresponse rate after being doubled to overcome the design effect [17]. Two-stage geographical cluster sampling was used to select the participants. For the first stage, all Grama Niladari (GN) areas (440) in Jaffna District were utilized as the sample framework, whereas people older than 60 years in each GN area were utilized as the sampling framework for the second stage. A total of 44 GN areas (10%) were selected from the first stage. Twenty elderly people from each of the selected GN areas were selected randomly in the second stage. Altogether, 880 elderly people were included in the study.

### Measures

The socio-demographic characteristics and economic factors were assessed using an interviewer-administered questionnaire. The QOL of the elderly was determined by the previously validated Tamil version of the WHOQOL-Bref questionnaire. This questionnaire consists of 26 questions. The questions were divided into four categories to assess the perceived QOL of the elderly in the following four domains; physical health domain, psychological domain, social participation domain, and environmental domain. Responses were obtained on a 5-point Likert scale. Individual scores for all four domains of quality of life were calculated using the transformed score table. A higher score indicates better QOL in all four domains in elderly individuals [16]. Data collection was conducted by face-to-face interviews at the participants’ homes. Informed written consent was obtained from the participants before the interviews. Recall bias was considered minimal, as the information gathered was highly personal.

### Analysis

The Statistical Package for Social Sciences (SPSS) for Windows, version 16, was used to analyses all the data collected. The results for sociodemographic and economic variables, and quality of life were presented using both descriptive and inferential statistics. For categorical variables, the findings are expressed as proportions and frequencies. The continuous variables’ standard deviation and mean are presented. The mean variations in the quality of life with the socio-economic factors were identified using one-way ANOVA and independent t-tests. Variables with a p-value less than 0.05 in the univariate analysis were included in the multiple linear regression models, and variable multicollinearity was examined before inclusion to the model. This model was used to examine the independent relationship of socio-economic characteristics with physical health, psychological, social participation, and environmental domains. A p-value less than 0.05 was considered statistically significant.

### Ethical consideration

The Ethics Review Committee of the Faculty of Medicine at the University of Jaffna in Sri Lanka granted ethical approval for this study (Ref. No-J/ERC/18/92/DR/0059). Informed written consent was obtained from all the participants in the study. Data was anonymised to protect confidentiality during analysis.

## Result

### Characteristics of participants

The mean age of the participants was 71.1years (range 60–100 years, SD 7.7). As shown in Table 1, of the 813 participants, 53.5% were men and 47% were in the 60–69 years age category. The higher proportion of participants were Hindus (85.9%). Most of the participants (61.6%) were having a spouse, and 74.7% had up to the secondary level of education. Less than half (47%) of the participants never went for work outside the home while others were currently working or retired from their employment. Nearly half (54.9%) of the participants had a monthly income below the national poverty line (food and non-food expenditures per person per month) of the region.

**Table 1:** Characteristics of the participants.

Sociodemographic Factors		F	%
Age (Years)			
	60–69 years	382	47.0
	≥70 years	431	53.0
Sex			
	Male	435	53.5
	Female	378	46.5
Religion (n = 797)			
	Hindu	685	85.9
	Christian	107	13.5
	Islam	05	0.6
Marital status (n = 811)			
	Unmarried	20	2.5
	With spouse	500	61.6
	widow	261	32.2
	Divorced/separated	30	3.7
Number of children			
	No	58	7.1
	1	49	6.0
	2–3	424	52.2
	More than 4	282	34.7
Education			
	No schooling	14	1.7
	Primary	98	12.1
	Secondary	607	74.7
	Collegial	58	7.1
	Tertiary	36	4.4
Resides with (n = 803)			
	Spouse only	254	31.6
	Children only	218	27.1
	Spouse and children	211	26.3
	Alone	95	11.8
	Others	25	3.1
Occupation			
	Retired	136	16.7
	Currently working	295	36.3
	Never worked	382	47.0
Monthly income (n = 699)			
	Below national poverty line	384	54.9
	Above national poverty line	315	45.1

### Univariate analysis

Univariate analysis revealed the participants’ levels of QOL in of different domains. Among the four QOL domains, the mean score for the environmental domain was higher (12.1±2.1) followed by psychological (2.0±2.8), physical health (11.8±2.3), and social participation domain (10.1±3.0).

### Bivariate analysis

Table 2 shows the individual associations of socio-economic characteristics with the four domains of QOL. All four domains of QOL were significantly associated with marital status, level of education, living arrangement, employment, source of income, level of income, income adequacy, and homeownership (P<0.05). Physical health, psychological, and social participation domains were significantly associated with age and gender (P<0.05). Religion was associated with the environmental domain (P = 0.001). The number of children was associated with the psychological, social relationship, and environmental domains (P<0.05). The presence of monthly income was associated with the psychological and environmental domains of the QOL (P<0.05).

**Table 2:** Associations of socio–economic characteristics with the four domains of QOL.

Socio-demographic factors		N	%	Physical domain Mean (SD)	Psychological domain Mean (SD)	Social relationship domain Mean(SD)	Environmental domain Mean (SD)
Age (in years)							
	60–69 years	382	47.0	12.5 (2.0)	12.3 (2.7)	10.9 (3.0)	12.2 (2.1)
	≥70 years	431	53.0	11.1 (2.3)	11.7 (2.8)	9.4 (2.9)	12.0 (2.1)
				P<0.001	P = 0.001	P<0.001	P = 0.279
Sex							
	Male	435	53.5	12.3 (2.2)	12.3 (2.7)	11.0 (2.9)	12.2 (2.0)
	Female	378	46.5	11.2 (2.3)	11.6 (2.8)	9.1 (2.9)	12.0 (2.1)
				P<0.001	P = 0.002	P<0.150	P<0.001
Religion							
	Hindu	685	85.9	11.8 (2.3)	12.0 (2.8)	10.1 (3.1)	12.2 (2.1)
	Christian	107	13.5	11.6 (2.4)	11.5 (2.8)	10.2 (2.6)	11.4 (2.2)
	Islam	05	0.6				
				P = 0.334	P = 0.053	P = 0.937	P = 0.001
Marital status							
	Unmarried	20	205	10.8 (2.7)	9.2 (2.7)	6.7 (1.9)	10.6 (2.2)
	Married/living with spouse	500	61.6	12.4 (2.1)	12.7 (2.6)	11.7 (2.6)	12.5 (2.0)
	Widowed	261	32.2	10.8 (2.1)	11.2 (2.6)	7.8 (1.8)	11.6 (2.1)
	Divorced/Separated	30	3.7	10.7 (1.8)	9.0 (2.3)	7.1 (1.0)	10.1 (2.1)
				P<0.001	P<0.001	P<0.001	P<0.001
Number of children							
	No	58	7.1	11.1 (2.0)	9.7 (2.4)	8.9 (2.8)	11.1 (1.9)
	1	49	6.0	11.6 (1.9)	11.2 (2.9)	10.2 (2.8)	12.0 (1.9)
	2–3	424	52.2	11.9 (2.1)	12.0 (2.6)	10.2 (2.8)	12.0 (1.9)
	More than 4	282	34.7	11.8 (2.7)	12.5 (2.9)	10.4 (3.4)	12.5 (2.3)
				P = 0.124	P<0.001	P<0.001	P<0.001
Education							
	No schooling	14	1.7	10.0 (3.3)	9.7 (2.8)	8.4 (4.9)	10.9 (3.5)
	Primary	98	12.1	11.0 (2.6)	11.2 (2.8)	8.7 (3.1)	11.3 (2.2)
	Secondary	607	74.7	11.7 (2.1)	11.8 (2.7)	10.1 (2.9)	12.0 (2.0)
	Collegiate	58	7.1	13.1 (1.7)	13.8 (2.2)	12.2 (2.6)	13.4 (1.8)
	Tertiary	36	4.4	13.6 (1.6)	14.2 (2.0)	11.8 (3.0)	14.4 (1.4)
				P<0.001	P<0.001	P<0.001	P<0.001
Resides with							
	Spouse only	254	31.6	12.3(2.1)	12.6(2.6)	11.8(2.1)	12.6(1.9)
	Children only	218	27.1	10.8(2.3)	11.5(2.6)	7.9(2.0)	11.8(2.0)
	Spouse and children	211	26.3	12.7(2.1)	12.9(2.4)	12.0(2.8)	12.6(1.9)
	Alone	95	11.8	11.2(2.1)	10.5(2.8)	7.4(1.7)	11.2(2.2)
	Others	25	3.1	10.2(2.1)	8.6(1.8)	6.9(1.9)	9.6(2.3)
				P<0.001	P<0.001	P<0.001	P<0.001
Occupation							
	Retired	136	16.7	13.1 (1.9)	13.7 (2.3)	11.9 (2.6)	13.8 (1.7)
	Currently working outside home	295	36.3	12.2 (2.0)	11.9 (2.7)	11.0 (2.8)	11.8 (1.8)
	Never worked outside home	382	47.0	11.0 (2.3)	11.4 (2.7)	8.9 (2.8)	11.7 (2.2)
				P<0.001	P<0.001	P<0.001	P<0.001
Presence of monthly income							
	Yes	676	83.8	11.8(2.3)	12.1(2.6)	10.2(3.0)	12.2(2.0)
	No	131	16.2	11.8(2.6)	11.3(3.2)	9.9(3.2)	11.3()2.6
				0.976	0.002	0.349	0.000
Level of monthly income							
	Below national poverty line	384	54.9	11.0(2.2)	10.9(2.4)	9.2(2.6)	11.3(1.7)
	Above national poverty line	315	45.1	12.8(2.2)	13.4(2.5)	11.5(3.1)	13.2(2.0)
				P <0.001	P <0.001	P <0.001	P <0.001
Income adequacy							
	Adequate	300	39.8	12.5(2.2)	13.4(2.5)	11.2(2.9)	13.2(1.7)
	Inadequate	454	60.2	11.3(2.2)	11.2(2.6)	9.5(2.9)	11.5(2.0)
				P<0.001	P<0.001	P<0.001	P<0.001
Living place							
	Own house	526	65.1	12.3(2.2)	12.5(2.6)	11.2(2.7)	12.5(1.8)
	Others house	265	32.8	10.7(2.2)	11.0(2.8)	8.1(2.4)	11.4(2.3)
	Self-rented house	17	2.1	11.0(1.6)	10.8(2.9)	8.5(2.9)	11.5(3.1)
				P <0.001	P <0.001	P <0.001	P <0.001

### Multivariate analysis

Tables 3–6 show the effect of various socio-economic factors on physical health, psychological, social participation, and environmental domains. Factors such as male sex, higher monthly income, income adequacy, and home ownership all contributed positively to the physical health domain. While being older and not having formal education had a negative impact on the physical health domain of QOL.

**Table 3:** Contributing factors of physical health domain of the QOL of participants.

Factors	B-	S.E	Beta	t	P- Value	95% CI B	
						Lower	Upper
(Constant)	9.187	.295		31.189	.000	8.608	9.765
Age 70	.951	.172	.201	5.530	.000	.613	1.289
Male sex	.560	.178	.118	3.143	.002	.210	.909
Having spouse	.429	.234	.089	1.838	.067	-.029	.888
Educated	.229	.237	.033	.965	.335	-.237	.695
Spouse and or children	.268	.255	.043	1.053	.293	-.232	.768
Working/worked	.297	.208	.062	1.431	.153	-.111	.706
Above national poverty line	.891	.182	.190	4.884	.000	.533	1.249
Adequate income	.726	.176	.153	4.125	.000	.380	1.072
House ownership	.486	.203	.096	2.387	.017	.086	.885

(Multiple linear regression R = 0.546, R^2^ = 0.298 Adjusted R^2^ = 0.288 SE = 1.971) Significance P<0.05 (Higher score indicate better QOL)

**Table 4:** Contributing factors of psychological domain of the QOL of participants.

Factors	B-	S.E	Beta	t	P- Value	95% CI B	
						Lower	Upper
(Constant)	8.278	.532		15.555	.000	7.233	9.323
Age 70	.589	.190	.106	3.107	.002	.217	.962
Male sex	.035	.196	.006	.180	.857	-.350	.421
Having spouse	.371	.261	.066	1.422	.155	-.141	.884
Educated	.183	.263	.022	.697	.486	-.333	.699
Spouse and or children	1.719	.292	.234	5.880	.000	1.145	2.293
Working/worked	-.361	.233	-.064	-1.549	.122	-.818	.097
Above national poverty line	1.511	.203	.275	7.459	.000	1.113	1.908
Adequate income	1.471	.194	.266	7.574	.000	1.090	1.852
House ownership	.651	.225	.110	2.888	.004	.208	1.093
Number of children	.207	.054	.126	3.839	.000	.101	.313
Having monthly income	-.483	.443	-.034	-1.089	.276	-1.352	.387

(Multiple linear regression R = 0.622, R^2^ = 0.387 Adjusted R^2^ = 0.377 SE = 2.158) Significance P<0.05 (Higher score indicate better QOL)

**Table 5:** Contributing factors of Social participation domain of the QOL of participants.

Factors	B-	S.E	Beta	t	P- Value	95% CI B	
						Lower	Upper
(Constant)	4.849	.328		14.766	.000	4.204	5.494
Age 70	.186	.176	.030	1.056	.291	-.160	.533
Having spouse	2.586	.236	.412	10.935	.000	2.121	3.050
Educated	.592	.244	.064	2.424	.016	.113	1.072
Spouse and or children	1.346	.271	.164	4.970	.000	.814	1.878
Working/worked	.605	.202	.096	2.991	.003	.208	1.002
Above national poverty line	.522	.188	.085	2.778	.006	.153	.892
Adequate income	1.225	.179	.198	6.828	.000	.873	1.577
House ownership	1.194	.209	.181	5.709	.000	.783	1.605
Number of children	.046	.050	.025	.914	.361	-.053	.144

(Multiple linear regression R = 0.753, R^2^ = 0.567 Adjusted R^2^ = 0.567 SE = 2.0238) Significance P<0.05 (Higher score indicate better QOL)

**Table 6:** Contributing factors of environmental domain of the QOL of participants.

Factors	B-	S.E	Beta	t	P- Value	95% CI B	
						Lower	Upper
(Constant)	8.278	.532		15.555	.000	7.233	9.323
Having spouse	.589	.190	.106	3.107	.002	.217	.962
Educated	.035	.196	.006	.180	.857	-.350	.421
Spouse and or children	.371	.261	.066	1.422	.155	-.141	.884
Working/worked	.183	.263	.022	.697	.486	-.333	.699
Above national poverty line	1.719	.292	.234	5.880	.000	1.145	2.293
Adequate income	-.361	.233	-.064	-1.549	.122	-.818	.097
House ownership	1.511	.203	.275	7.459	.000	1.113	1.908
Number of children	1.471	.194	.266	7.574	.000	1.090	1.852
Male sex	.651	.225	.110	2.888	.004	.208	1.093
Hindu religion	.207	.054	.126	3.839	.000	.101	.313
Having monthly income	-.483	.443	-.034	-1.089	.276	-1.352	.387

(Multiple linear regression R = 0.618, R^2^ = 0.382 Adjusted R^2^ = 0.371 SE = 1.971) Significance P<0.05 (Higher score indicate better QOL)

In regards to the psychological domain of QOL, having more children, living with a spouse and or children, higher monthly income perceived income adequacy and ownership of house were positively contributed to the psychological domain of the QOL. The following factors negatively contributed to the psychological domain of QOL: higher age, not having formal education.

The social relationship domain was positively contributed by the factors such as male sex, having a spouse, living with spouse and or children, higher monthly income, income adequacy and ownership of a house, while negatively contributed by higher age, never going to work outside the home.

The environmental health domain of QOL was positively contributed by a higher number of children, living with spouses and or children, higher monthly income, income adequacy, and ownership of a house, while not having formal education was negatively contributed.

## Discussion

The scores of all four domains of QOL of the elderly in Jaffna district were lower than the global average of WHOQOL-bref scores of the elderly age group [18]. Sri Lanka faced three decades of civil war, which ended in 2009, Jaffna district was one of the most affected places by the war. The war-damaged the Jaffna community in all physical, psychological, social, and environmental dimensions. The lowest QOL of the elderly in the current study might be the residual effect of the said war. Further, the proportion of chronic diseases is high in the Jaffna district [19]. Chronic diseases might be a reason for the reduced physical health domains of the QOL.

Results show that the social participation domain had the lowest mean score in this study. The traditional social support system is being overwhelmed by the rapid expansion of the elderly population in developing countries [20]. As a result, the elderly have become a neglected population category. This could be one of the reasons for the lowest social participation among the elderly in the current study. Furthermore, deaths of close ones, as well as property losses due to the war [21] might lead to the social participation domain of QOL being the lowest of the four domains. The current finding is supported by the studies conducted in India [20] and Bangladesh [22], where the lowest domain score of QOL was reported for the social participation domain.

In the current study, age was found to be strongly associated with all domains of QOL except the elderly’s environmental domain. Similar findings have been observed in research undertaken in Sri Lanka [12] and other countries [23–26]. Dependency of the elderly increases as they get older [26], which may contribute to the lower QOL with the increase in age. When compared to the two age categories, the elderly who are young-elderly (age below 70 years) had the highest QOL in all domains. Even though physical health can be impaired with aging [27], the young elderly are still in the early stages of physical deterioration. They can carry out their daily activities more independently than elderly with higher age. This might increases their QOL. Furthermore, as one gets older, the risks of losing a spouse and being separated from one’s children rise. In the current study, the risk of living alone is higher among the elderly aged over 70 years compared to the elderly aged below 70 years of age (ODD 1.788 CL: 1.194–2.678), which can have a detrimental impact on one’s quality of life. These factors might contribute to the Higher QOL of the young elderly than their counterparts.

In the current study, males perceived higher QOL than females in all domains except the environmental domain. A prior study conducted in Jaffna supported the present finding [13]. Further research was carried out in Qatar [28], Lebanon [29], India [20, 30], Bangladesh [31], Vietnam [32] Indonesia [29] Malaysia [33], stating that gender has a substantial impact on QOL, with females experiencing lower QOL than males. The countries indicated above in Southeast Asia and the Middle East adhere to some traditional gender ideals, which influence male and female role performance in society which might contribute to the difference in QOL with gender. It can be noted that there was no gender difference in QOL was reported in the studies conducted in Japan [34] and Thailand [35]. These inconsistencies may be associate with the differences in cultural values surrounding gender in different countries. Jaffna society was known as a culturally enriched society with a higher ideological linage which may explain the gender differences in the perception of QOL of the elderly in the current study and reflect the similar pattern of its associated neighbouring countries.

Only the environmental domain scores showed significant differences between the religious groups in the current study. The elderly engage in more religious activities than other age groups [36] to cope with the challenges associated with aging [37]. Religion was known to play a significant role in the lives of the elderly Sri Lankans [12]. Practices and accompanying culture were significant among the three religious communities of this study, but they were not reflected in their QOL. This can be explained by the fact that the level of religious belief and faith may be linked to QOL, but religion itself does not alter the QOL of the elderly.

All four QOL domains of elderly QOL were found to be significantly associated with the marital status of the elderly. The majority of the elderly in the current study had spouses (61.6%). They reported higher QOL when compared to the unmarried, widowed, or divorced/separate elderly. Prior studies in Sri Lanka [13, 38] and other countries had found that married status was linked to elderly people’s quality of life [20, 23, 26, 30]. It was found that people with spouses had better mental and physical health and lived longer than the people without spouses [39, 40, 41]. Also the elderly with a spouse can share and alleviate their partner’s distress. Furthermore, having a spouse ensures the receipt of care and avoids loneliness. In addition to that, having a healthy relationship with one’s spouse will improve one’s mental health [42]. These factors might contribute to the higher QOL of the elderly who have a spouse than those who don’t have a spouse.

The number of children of the elderly was significantly associated with all three QOL domains except for the physical health domain in the present study. QOL was highest among the elderly with more than four children. It was reported in a previous study that the elderly look at the children as resources for their old age [43]. Having more than four children [43], and living in a large family [32] also contributed to the elderly’s better QOL. A Sri Lankan study reported that the elderly are respected by their children as a good traditional practice [5]. Also, elderly people with more children were found to have enjoyed better living conditions, received more monetary assistance, and had more providers for in-kind and emotional care in Sri Lanka [5]. This may have contributed positively to pursuing a good QOL for the elderly. Jaffna society is also a traditional society with a higher emotional bond with their children. Having more children is associated with better QOL among the elderly in the current study.

The level of education was found to be significantly related to all four domains of QOL of the elderly in the current study. Several previous studies have also found a positive relationship between education level and on the perception of QOL among the elderly [12, 31, 41, 44] Education is a determinant of many other elements in an individual’s life, including occupation, income, living arrangement, property ownership, and social capital. It can have both a direct and indirect effect on an individual’s QOL. According to other researchers, people with a higher level of education are more likely to live a healthy lifestyle [45, 46], have better problem-solving abilities [22], are happier, have strong social relationships, and have better self-assessed health [47, 48]. This explains why the elderly with higher educational levels perceive higher QOL compared to those who have a lower level of education.

The majority of the elderly live with either their spouse or children or both in the current study. The elderly who reside with their spouse and or children perceive significantly higher QOL in all the domains, compared to the elderly who live alone or with other living arrangements. This finding was supported by several researchers in Sri Lanka [12], and other countries [20, 23, 26, 31, 32, 49]. Elderly people who live with their spouse and children are more likely to receive better care and emotional support, which could explain the current study’s findings.

Around one-third of the elderly (36.3%) were currently working, and around half of the elderly (47.0%) were unemployed while 16.7% of them were retired from their work. Further employment status was significantly associated with all the domains of QOL of the elderly in the current study. The highest mean QOL was perceived by the elderly who were retired from previous employment. This finding was supported by several studies [13, 30, 31, 50, 51]. Even though the elderly who are currently working can have good physical functioning and social contact compared to the elderly who are retired, they perceive comparably low QOL. Most of the elderly (94.8%) who were retired from their previous employment were pensioners in the present study. This can ensure financial independence which may contribute to their higher QOL. Also, the elderly who were never going to work scored the lowest QOL in all domains compared to the currently working and retired elderly. This may be associated with financial dependency and less interaction with their society.

A considerable proportion of participants (83.8%) had a consistent monthly income. A very higher proportion (83.8%) of respondents had a regular monthly income. It was significantly associated only with the psychological and environmental domains of QOL of the elderly. In other studies [52], the presence of a monthly income was identified as an associated factor of QOL for the elderly. While some researchers stated that it was not associated with the QOL of the elderly [53], as the level of income, income source, and wealth management abilities were important factors that contributed to the perception of QOL more than having a regular monthly income [53, 54].

Previous studies [55] have proven that economic independence was associated with higher QOL among the elderly. A higher level of income ensures that basic needs are met, that social participation and social respect are maintained, and that the elderly are not concerned about unexpected healthcare expenses, thereby improving their QOL. In the current study, income adequacy was significantly associated with all domains of the QOL of the elderly. It was reported as a direct contributing factor of QOL in the studies conducted in Sri Lanka [12, 13] and other countries [24, 25, 31, 50]. Income adequacy ensures a sense of financial security and a sense of independence from the needs of others. Perceived adequateness of income can reduce the fear of health care costs which may increase the QOL of the elderly.

The elderly who own a house had significantly higher QOL in all domains than those who live in other houses. This finding was supported by other researchers [15, 31, 32, 53]. The elderly prefer to live in their own houses to preserve their authority in the family and to maintain the provider role. It was reported that ownership of the house had a marked impact on the receipt of care of the elderly from their family members [15]. Thus, ownership of a house may ensure dignity and increase the probability of receiving better care for the elderly. These together may be contribute to the higher QOL of the elderly who live in their own house than the elderly who live in other houses.

## Conclusion and recommendation

The QOL of the elderly in the Jaffna district of Sri Lanka is low. The environment domain had the higher score, while the social health domain had the lowest score in this study. Marital status, level of education, living arrangement, employment status, source of income, level of income, income adequacy, and ownership of a house were associated with all the domains of QOL of the elderly.

Male gender, having spouse, number of children, living with spouse and or children, presence of monthly income, income adequacy, and ownership of house were positively contributed at least one domain of QOL of elderly. While, being older, in the current study of primary education, never went for work outside home, had a negative impact on at least one domain of QOL of elderly. Interventions to improve the QOL of the elderly are expected. When planning such interventions, unique factors such as the number of children, living arrangements, and ownership of house should be considered in addition to the known contributing factors to the QOL of the elderly. Also Interventions that place great emphasis on social support and social participation of elderly are recommended.

## Supporting information

S1 Data (SAV)
